# Safety in selective surgical exploration in penetrating neck trauma

**DOI:** 10.1186/s13017-016-0091-4

**Published:** 2016-07-12

**Authors:** Frederico Teixeira, Carlos Augusto Metidieri Menegozzo, Sérgio Dias do Couto Netto, Renato S. Poggeti, Francisco de Sales Collet e Silva, Dario Birolini, Celso de Oliveira Bernini, Edivaldo Massazo Utiyama

**Affiliations:** Departament of Surgery, Division of Surgical Clinic III, University of Sao Paulo School of Medicine, Sao Paulo, Brazil

**Keywords:** Neck injury, Penetrating trauma, Cervical trauma, Surgical selective management

## Abstract

**Background:**

Selective management of penetrating neck injuries has been considered the standard of care with minimal risks to patient safety. In a previous non-randomized prospective study conducted at our center, selective management proved to be safe and reduced unnecessary exploratory cervicotomies. In the present study, the role of clinical examination and selective diagnostic tests were assessed by reviewing demographic and clinical data. A comparison of results between two groups (mandatory surgical exploration versus selective surgical exploration) was made to check the safety of selective management in terms of the rates of morbidity and mortality.

**Methods:**

A retrospective analysis at the Emergency Department of the Hospital das Clínicas of the University of Sao Paulo was performed by a chart review of our trauma registry, identifying 161 penetrating neck trauma victims.

**Results:**

Of the 161 patients, 81.6 % were stabbed and 18.4 % had gunshot injuries. Stratifying the wound entry points by neck zones, we observed that zone I was penetrated in 32.8 %, zone II in 44.1 % and zone III in 23.1 % of all the cases. Thirty one patients (19.2 %) had immediate surgical exploration, which had a mean length of stay of 6 days, a complication rate of 12.9 % and a mortality rate of 9.4 %. Of the 130 who underwent selective surgical exploration 34 (26.1 %) required operative procedures after careful physical examination and diagnostic testing based on clinical indications. The mean length of stay for the selective surgical exploration group was 2 days with a complication rate of 17.6 % with no mortality, and virtually all of them were related to associated injuries in distant body segment. No statistical significance was found comparing mortality and complication rates between the two groups. Selective approach avoided 59 % of unnecessary exploratory cervicotomies.

**Conclusion:**

Careful evaluation of asymptomatic and stable patients with minor signs of injury can safely avoid unnecessary neck explorations with low rates of morbidity. This should be the standard management of such patients.

## Background

One of the most important characteristics of the neck is the anatomical relationship between vital structures and their relative vulnerability to injuries. Penetrating neck injuries (PNI) can result in simultaneous lesions in the aerodigestive and respiratory tracts, vertebral column and calvarium, blood vessels and lymphatic ducts. For several years, especially due to the military experience in the World War II, penetrating neck injuries were treated with exploratory surgery and this was recognized as the optimal management [1, 2]. The rationale for this approach was based on morbidity and mortality associated with missed injuries, although resulted in a high rate of non-therapeutic cervicotomies [1, 3–5].

In the civilian population, the mandatory exploration policy was questioned in the past two decades with a number of prospective series in the literature reporting the usefulness of the selective management of penetrating neck injuries [3–6]. In a previous non-randomized prospective study including 53 patients with penetrating neck injuries admitted to our emergency departament, selective surgical approach safely reduced the rate of unnecessary exploratory cervicotomies [7].

On the other hand, many surgeons prefer selective surgical exploration based on mandatory use of diagnostic studies which can be expensive and not readily available, especially in most Latin American emergency departments [5, 8, 9]. Therefore, we performed a retrospective analysis of PNI management in an urban trauma center in Brazil. Our objective was to assess the safety of the selective approach. Our main outcomes included morbidity and mortality rates. Our secondary outcome was the avoidance of unnecessary cervicotomies.

## Methods

A chart review of enetrating neck trauma patients was done in order to evaluate our experience. All patients who presented with penetrating neck injuries met the inclusion criteria. Exclusion criteria included superficial wounds, defined by injuries superficial to the plane of the platysma muscle, and death before admission. Demographic data, pre - hospital care, mechanism of injury, Revised Trauma Score (RTS) and Injury Severity Score (ISS) were obtained from the Emergency Department trauma registry.

All patients were initially assessed according to Advanced Trauma Life Support guidelines by the attending trauma surgical staff, led by an experienced trauma surgeon consultant. The site of injury was classified using anatomical landmarks: Zone I extending from the base of the neck to the cricoid cartilage; Zone II is the area between the cricoid and the inferior border of the mandible; and Zone III comprising the area between the angle of the mandible to the base of the skull [10]. First physical exam meant to divide these patients into two categories. The first category included those presenting with hard signsand symptoms [6, 9] of aerodigestive severe injuries such as bubbling, hoarseness, salivary fistula, expanding hematomas, profuse bleeding or hemodynamic instability; these patients underwent mandatory exploration. On the other hand, hemodynamically stable patients with soft signs and symptoms [6, 9] such as dysphagia, crepitation, stable hematoma, hemoptysis or hematemesis, were treated with the selective approach. Patients with suspected spinal cord injury had neurosurgical consultation, and the decision of surgical repair of these injuries was at the discretion of the neurosurgical team. Assympotmatic patients were physically evaluated, with local exploration, for the depth of the injury by the consultant surgeon. If a superficial wound was identified, simple suturing was performed, followed by hospital discharge.

Neck Computed Tomography was utilized in cases of suspected cervical fractures, spinal cord injuries or in the setting of concomitant severe facial or head injury. Esophagoscopy, laryngoscopy, bronchoscopy and swallow studies were performed based on trauma topography, presence of signs and symptoms suggestive of aerodigestive tract or subcutaneous emphysema signs on radiographic studies. Profuse bleeding from neck injury was initially managed with balloon tamponade [11]. Stable hematomas, audible bruits, palpable thrill or abnormal pulses in Zone II injuries were investigated using duplex scan. Angiographic studies were only performed in Zone I and III injuries with suspicious clinical findings, or uncertain duplex scan images of Zone II injuries.

All asymptomatic patients were observed for a minimum of 24 h. The surgical technique used was a vertical incision on the anterior border of the sternocleidomastoid muscle for Zone II and median sternotomy for Zone I injuries. Post auricular extension of the vertical incision was performed for distal vascular control in Zone III injuries. Occasionally bilateral vertical incisions were required for transfixating cervical lesions.

Statistical analysis and comparison was performed using chi-square and t-student tests using SPSS Statistics® software. A *p* value < 0.05 was considered statistically significant.

## Results

During a five-year period, 181 patients were admitted with stab or gunshot injuries in the neck. Of those patients, 161 presented with neck wounds violating the platysma. Demographic information of the admitted patients is shown in Table 1. The time of arrival ranged from 15 min to 120 min, with a mean of 35 min. Of the gunshot injuries 17 (57 %) were considered transcervical. In 6 (3.4 %) stabbed patients the wound was created by mirrors, screw divers, razors and in one case by a circular saw. Surgical neck exploration was the treatment of choice for 65 (40 %) patients.

**Table 1:** Admission data of the 161 patients presenting with penetrating neck injuries

	Number of patients (%)
Gender	
Male	143 (88 %)
Female	18 (12 %)
Mean Age (years)	26
Pre-hospital care	
Land Unit	143 (89 %)
Aeromedic Unit	2 (1 %)
Other	16 (10 %)
Mechanism of Injury	
Gunshot Wound (GSW)	29 (18 %)
Stab Wound	
Knife	126 (78 %)
Other	6 (4 %)
Injury Location	
Zone I	53 (33 %
Zone II	71 (44 %)
Zone III	37 (23 %)
Management	
Selective Approach	130 (81 %)
Mandatory Surgery	31 (19 %)

Thirty one patients admitted having hard signs of vascular or aerodigestive injuries were immediately taken to the operative room. The mechanism of injury was gunshot in 9 (29 %) and stabbing in 22 (71 %) cases.

In the mandatory neck exploration group, 8 (25.8 %) had Zone I injuries, 17 (54.8 %) Zone II injuries, and 6 (19.4 %) Zone III injuries. Of them, 7 (22.6 %) had major vascular injuries such as jugular, carotid and vertebral vessels, and 24 (77.4 %) had injuries on the trachea and larynx, esophagus or pharynx. The signs and symptoms which indicated immediate neck exploration are shown in Table 2.

**Table 2:** Mandatory surgery group: clinical findings

	Number of patients (%)
Bleeding	20 (64 %)
Expanding Hematoma	7 (22 %)
Bubbling	2 (6 %)
Hard Aerodigestive Symptoms	1 (3 %)
Persistent hemorrhagic shock	1 (3 %)

Neck balloon tamponade was required in one patient, who presented with major vascular injury active bleeding in the oral cavity. One patient presented with profound shock and recalcitrant coagulopathy secondary to a carotid injury. Damage control with temporary arterial shunt was necessary in that case.

Table 3 displays data about the patients of the Selective Approach Group. All of the stable patients who presented signs or symptoms of vascular injuries were investigated with duplex ultrasound for Zone II injuries and angiography for Zone I or Zone III injuries.

**Table 3:** Selective approach group (*n* = 130)

	Number of patients (%)
Observation only	96 (74 %)
Surgical Exploration	34 (26 %)
Clinical Findings	
None	39 (30 %)
Bleeding	47 (36 %)
Disphagia or odinophagia	11 (8 %)
Stable Hematoma	10 (8 %)
Hoarseness	9 (7 %)
Hemoptysis	7 (5 %)
Emphysema	6 (5 %)
Stridor	1 (1 %)
Surgical Findings	
Pharingoesophageal Injury	10 (29 %)
Laringotracheal Injury	21 (62 %)
Combined Aerodigestive Injury	3 (9 %)

Fifty-four (41,5 %) patients had Zone II injuries and all of them underwent duplex ultrasound investigation. Angiography was performed in 16 (12.3 %) patients, with 6 Zone I and 9 had Zone III injuries. In only one case of Zone II injury, an angiography was performed due an inconclusive duplex ultrasound. Diagnosis of vascular injuries were made in 9 (13 %) stable patients by duplex ultrasound or angiography. All patients with minor vascular injuries, except one, were expectantly managed and no surgical exploration was necessary. In one patient a vertebral artery injury was identified. Selective embolization of the artery was performed to control of the bleeding.

The patients with signs and symptoms of aerodigestive tract injuries were submitted to diagnostic investigation with esophagoscopy, laryngoscopy and bronchoscopy. In 34 patients, the diagnostic investigation with endoscopic tests indicated surgical exploration. Of them, 30 (88.2 %) were stabbed and 4 (11.8 %) had gunshot wounds. In the group of patients submitted to selective neck explorations, 7 (20.6 %) had Zone I injuries, 19 (55.9 %) had Zone II injuries and 8 (23.5 %) had Zone III injuries.

The trauma scores, morbidity and mortality of both groups are shown in Table 4. Mortality was observed only in the mandatory surgical exploration group. All 39 patients with no clinical findings were observed for at least 24 h and discharged without evidence of clinical compromise. A Selective management policy avoided non-therapeutic neck explorations in 59.6 % of the patients, as these patients had uncomplicated clinical courses with conservative management.

**Table 4:** Trauma scores and complications

	Selective approach (*n* = 130)	Mandatory surgery (*n* = 31)	
ISS (mean)	17	26	
RTS (mean)	7.62	5.83	
Complications			*p = 0.6*
Surgical site infection	2	2	
Haematoma	1	1	
Wound dehiscence	2	0	
Laryngeal recurrent injury	1	0	
Pharyngoesophageal fistulae	0	1	
Mortality	0	3	*P = 0.06*

## Discussion

The neck is a vulnerable area containing different vital organs of multiple physiological systems. Due to proximity of these anatomical structures, there is a high predisposition of multisystemic injuries with potentially life-threatening lesions of the laringotracheal and pharingoesophageal complexes, major blood vessels, spinal cord and cervical nerves. Mandatory surgical exploration was the mainstay form of treatment during past few decades, mostly because of this singular topographic characteristic. Moreover, reported cases of unrecognized neurovascular injuries, resulting from conservative treatment, prompted the widespread acceptance of mandatory surgical exploration for all injuries penetrating the platysma [12–18].

However, studies documenting the civilian experience in the management of penetrating neck injuries during the last 3 decades have shown high rates of non-therapeutic exploratory cervicotomies. As a consequence, Selective Approach of penetrating neck injuries based on clinical criteria and additional radiographic and endoscopic tests has emerged as an alternative management in several centers [19–31].

Neck injuries can represent a fair share of the trauma admissions in high-volume centers [4, 9], and the number of patients with neck trauma is rising in some countries, such as England [32]. Recent publications have highlighted the ongoing controversy about role of surgical exploration in the management of penetrating neck injuries [6, 24, 33–35].

Based on their cumulative experiences, busy trauma centers often adopt a policy of selective management for patients suffering PNI mainly based on clinical investigations and protocols [9, 20, 22, 24]. On the other hand, centers with limited number of admissions have a higher threshold for adjunctive investigation studies and favor the old policy of mandatory surgical exploration [36–38]. In fact, some studies promoted the mandatory exploration policy given the low morbidity and shorter length of stay [38]. This approach was stated as cost-effective and more accurate than imaging studies, especially endoscopic and contrast studies [39–41]. However, a publication from Nason et. al. showed no improvement in terms of hospital stay or morbidity in patients treated with mandatory exploration of penetrating neck injuries [5].

After analyzing a previous non-randomized prospective study validating the selective management of penetrating cervical injuries in our Trauma Service [7], we conducted a restrospective chart review of our patients who had PNI. Our goal was to obtain a realistic panorama of the assessment and management of patients admitted in a busy trauma center in Latin America with a larger cohort of patients.

The major indication for immediate surgery was active bleeding and expansive hematoma which comprises 87 % of surgical explorations in this group. In the selective management group 100 % of the injuries responsible for surgical explorations were in the aerodigestive tract. Adjunctive tests were indicated by judicious clinical examination based on the evolving concept that routine use of angiography and endoscopy in penetrating neck injuries patients rarely change the management [20, 22, 42, 43]. Demetriades et. al. found that the absence of clinical signs accurately excluded significant vascular and aerodigestive tract lesions [9]. Other studies show similar results regarding the safety of selective diagnostic testing [4, 23].

In this study, angiography was seldom indicated in the selective group and was almost restricted to Zones I and III injuries when clinical signs of vascular injuries were identified by detailed physical examination. Doppler examination is readily available and frequently performed at our institution making this noninvasive method of investigation useful when vascular injuries were suspected. An angiography was indicated in only one Zone II injury with an inconclusive duplex ultrasound result. Angiography was particular useful in a rare case of vertebral artery injury where therapeutic selective embolization was performed controlling the active bleeding. Since there is a reported complication in 0.2 % to 2 % of the catheter angiographies, some studies suggest the use of a safer, less costly and non-invasive diagnostic method such as duplex ultrasound, with similar accuracy [5, 9, 12]. Moreover, ultrasound is more widely available in trauma services.

Table 5 summarizes the accuracy of diagnostic studies in identifying lesions that required treatment, and includes data from other studies [4, 9, 44, 45]. The main difference between our results and the published literature lies in the positive predictive value (PPV) of endoscopic studies. This is mainly because our approach was to surgically explore lesions detected by these studies, both to confirm it´s extent and also to repair or drain the injury site. Nevertheless, we recognize reports of nonoperative management of small aerodigestive lesions [4, 9, 42]. With vascular injuries, interestingly there was just one patient that required intervention after diagnostic evaluation. As such, when analyzing test accuracy for identifying lesions that require some form of intervention, one will note that doppler and angiography show low PPV and high NPV, which confirms their value in excluding significant lesions. Although high NPV has been previously shown, our low PPV differs from the published literature [4, 9, 44]. We consider this to be possibly related to selection bias and lack of standardization in test reports, and might not reflect the real PPV of these tests.

**Table 5:** Diagnostic accuracy in identifying lesions that required treatment

	PPV	NPV
Doppler	20 % [44], 100 % [9] Teixeira F et al. 12 %	100 % [9, 44], Teixeira F et al. 100 %
Angiography	80 % [4], 73 % [9], 18 % [44] Teixeira F et al. 12 %	100 % [4, 9, 44], Teixeira F et al. 100 %
Endoscopic (aeordigestive)	20 % [4, 9, 42], 37,5 % [45], Teixeira F et al. 100 %	100 % [4, 9, 42, 45], Teixeira F et al. 100 %

*PPV* positive predictive value, *NPV* negative predictive value

We do recognize that more recent publications advocate the use of Computerized Tomography Angiography (CTA) for the evaluation of penetrating neck injuries, showing good accuracy for vascular and aerodigestive lesions [6, 22]. Although not available in a great number of institutions and posing the risks inherent to the use of contrast, this modality offers timely, safe and accurate evaluation of major aspects of neck injuries.

**Fig. 1:**
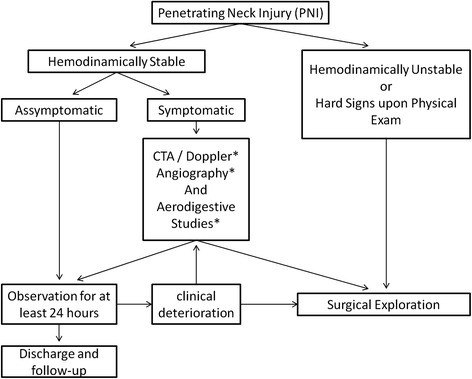
Management Algorithm. *diagnosic studies should be used according to available resources and experience in each center

Our retrospective analysis involved the management of patients prior to the adoption of that diagnostic tool as a routine practice in these patients in our service. As mentioned above, studies have showed that doppler ultrasound and endoscopic studies are accurate [5, 9, 12, 24, 42, 45] and maybe more available in some hospitals than CTA. Also, Thoma et al. published a prospective study in which complementary diagnostic studies were used based on physical examination findings and no CTA was performed, showing good results [4]. Hence, we believe that the present study could be used also for centers with limited resources, and that our results can help guiding management in these hospitals. Based in our results and the published data, we present an algorithm for management of PNI patients (Fig. 1).

When comparing results between mandatory and selective exploratory groups, postoperative morbidity and mortality were not significantly different between the groups. We found a complication rate of 4.5 % and 12.9 % in patients of selective approach and mandatory exploration groups, respectively. Other studies found lower rates of complication for surgically managed patients [5, 14, 39]. Mortality was observed only in mandatory surgery group (9.6 %), which could be partially explained by the patients´ higher ISS and RTS values at admission in that group. Solimann et. al. found similar results, while other groups report a lower mortality rate [4, 9, 31]. The mean time of hospitalization was lower in the selective approach group.

An important finding from our study is that almost 60 % of the patients were not submitted to non-therapeutic neck explorations, and no missed injuries were observed at follow up of selective managed patients. This is consistent with other studies showing avoidance of unnecessary cervicotomies in 30–89 % [4, 5, 37–40]. Low failure rates of selective managed patients have been reported, ranging from 0 to 3 % [4, 9, 28, 31]. Also in concordance with these results, none of the patients selectively managed with observation died or had to undergo surgery.

Our study is limited by all aspects inherent to a retrospective analysis. Since patients were managed based on individualized criteria, not every patient underwent full diagnostic work-up. Therefore, it is reasonable to recognize that clinically non-relevant injuries may have been missed. Also, there was lack of uniformity concerning the operative technique description and diagnostic tests reports which could limit the interpretation of the results.

## Conclusion

Management of patients with penetrating neck injuries is still challenging. Major signs and symptoms of vascular or aerodigestive injuries should prompt emergent surgical exploration. Our results further support previous evidence that selective management based in physical evaluation and diagnostic studies is safe and feasible in stable patients. These patients should be managed according to staff experience and resource availability. A selective approach is feasable and safely decreases the incidence of non-therapeutic cervicotomies and their complications.

## Abbreviations

CTA, computerized tomography angiography; HCFMUSP, hospital das Clinicas of the University of Sao Paulo School of Medicine; ISS, injury severity score; NPV, begative predictive value; PNI, penetrating neck injury; PPV, positive predictive value; RTS, revised trauma score
